# Monocytes in HIV and SIV Infection and Aging: Implications for Inflamm-Aging and Accelerated Aging

**DOI:** 10.3390/v14020409

**Published:** 2022-02-17

**Authors:** Zoey K. Wallis, Kenneth C. Williams

**Affiliations:** Department of Biology, Boston College, Chestnut Hill, MA 02467, USA; zoey.wallis@bc.edu

**Keywords:** HIV/SIV, aging, monocytes, macrophage, inflamm-aging

## Abstract

Before the antiretroviral therapy (ART) era, people living with HIV (PLWH) experienced complications due to AIDS more so than aging. With ART and the extended lifespan of PLWH, HIV comorbidities also include aging—most likely due to accelerated aging—as well as a cardiovascular, neurocognitive disorders, lung and kidney disease, and malignancies. The broad evidence suggests that HIV with ART is associated with accentuated aging, and that the age-related comorbidities occur earlier, due in part to chronic immune activation, co-infections, and possibly the effects of ART alone. Normally the immune system undergoes alterations of lymphocyte and monocyte populations with aging, that include diminished naïve T- and B-lymphocyte numbers, a reliance on memory lymphocytes, and a skewed production of myeloid cells leading to age-related inflammation, termed “inflamm-aging”. Specifically, absolute numbers and relative proportions of monocytes and monocyte subpopulations are skewed with age along with myeloid mitochondrial dysfunction, resulting in increased accumulation of reactive oxygen species (ROS). Additionally, an increase in biomarkers of myeloid activation (IL-6, sCD14, and sCD163) occurs with chronic HIV infection and with age, and may contribute to immunosenescence. Chronic HIV infection accelerates aging; meanwhile, ART treatment may slow age-related acceleration, but is not sufficient to stop aging or age-related comorbidities. Overall, a better understanding of the mechanisms behind accentuated aging with HIV and the effects of myeloid activation and turnover is needed for future therapies.

## 1. Introduction

Antiretroviral therapy (ART) has enabled people living with HIV (PLWH) to live longer, yet a high risk remains of developing HIV- and cumulative age-associated comorbidities. In 2018, the Center for Disease Control (CDC) reported that over half (51%) of people living with HIV in the United States were age 50 or older [1,2]. This aging group of PLWH stems from a larger aging population, with an estimated 12.5% increase in the number of people over the age of 65 in the next 20 years [1,2]. Although not well-understood, aging leads to alterations in the immune system and responses to HIV infection and coinfections. This review focuses on the biology of monocytes and macrophages with HIV and simian immunodeficiency virus (SIV) infection, and HIV infection and aging.

Monocytes and macrophages are important components of the innate immune system that link innate immunity with acquired immunity. They are involved in the first line of defense against pathogens, including retroviruses such as HIV and SIV and their clearance, as well as toning the immune response to balance tissue inflammation, injury, and repair. Monocytes are derived from the bone marrow (BM) as well as the fetal liver and spleen, and can replace tissue macrophages under normal physiologic conditions, during inflammation, and with pathology [3,4]. Within the central nervous system (CNS) there normally exist four to five macrophage populations, and the cardiovascular system and heart have several subpopulations, all of which change with aging, HIV, and disease [5,6]. Resident macrophages can be replaced by the recruitment of inflammatory monocytes from blood, as well as repopulation within the organ [5,6]. Resident tissue-macrophage populations are yolk-sack-derived and present in tissues at the embryonic stage and at birth. BM-derived monocytes, monocyte-derived macrophages, and yolk-sack-derived macrophages are diverse phenotypically and physiologically in different tissues [3,4]. These cells are thought to be repopulated in normal physiology at a rate that is augmented with HIV and SIV infection, inflammatory responses, and aging. Monocyte and monocyte/macrophages as well as resident tissue macrophages are critical in immune responses to retroviruses and by definition can be infected by them, but also play important roles in amplifying and toning immune responses, mediating lesion resolution, wound healing and repair [7,8]. With continued long-term immune stimulation by HIV and SIV in response to cell and tissue injury and death signals, and most importantly aging, these cells contribute to chronic immune activation termed “inflamm-aging”, seen with aging and HIV [9,10,11,12]. This review focuses on the biology of monocytes—and sometimes macrophages—with HIV infection and aging, and how these somewhat contradictory responses are not well-understood but are likely critical in aging populations with HIV and AIDS. Many of the studies described in this review are from the results of SIV infection of rhesus macaques. SIV is the premier model of HIV infection because of the genetic similarity of the virus, SIV’s defined tropism for myeloid cells, dendritic cells, and lymphocytes, and the similarities on pathogenesis of SIV-infected monkeys with AIDS in lymph nodes, blood, and tissues including the CNS and cardiovascular system [5,13,14]. It is interesting to note that SIV has the accessory gene Vpx in place of Vpr in HIV, which can result in a higher level of monocyte and macrophage infection [15]. SIV has similar stages of plasma viral expansion in plasma and tissues, and a latent period of infection. Importantly, antiretroviral agents used in HIV-infected humans are used in SIV-infected monkeys where ART leads to non-detectable plasma and tissue virus. Some of the pioneering work on monocyte and macrophage infection and their role in viral traffic and CNS and cardiac pathology have come from studies in SIV-infected monkeys [13,14,16,17,18].

## 2. Monocytes and Macrophages in HIV and SIV Infection and Aging

**Figure 1:**
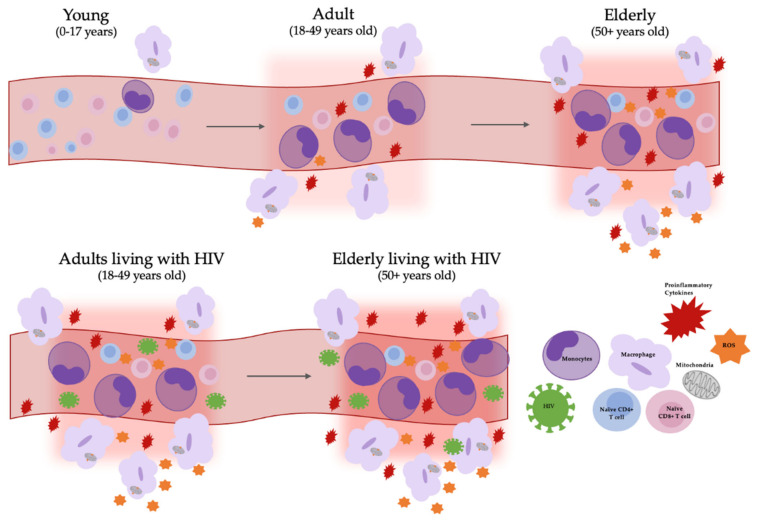
Altered myeloid production with aging and HIV infection contributes to chronic inflammation. Aging monocyte populations are skewed towards a proinflammatory phenotype and there is an overall decline in naïve CD4+ and CD8+ lymphocyte production [31,32,33,34]. Additionally, HIV/SIV infection causes an increase in the rate of production and turnover of myeloid cells [13,16,17,18,19,20,21]. Age induced-mitochondrial dysfunction increases reactive oxygen species (ROS) which also contributes to tissue inflammation [35,36,37,38]. Elderly adults and adults living with HIV both have persistent inflammation caused by myeloid cells in response to chronic immune activation.

With normal physiology in humans and non-human primates, monocytes comprise 2–10% of total white blood cells (WBC) in the blood. They are continually produced via BM hematopoiesis by hematopoietic stem cells (HSC) [19,20] (Figure 1). The kinetics in the blood of the different monocyte populations are distinct [21,22,23,24], where the rate of production and turnover are increased with HIV and SIV infection, aging, and HIV and aging [13,19,23,25,26,27,28]. He et al. found the production and differentiation of HSCs and monocytes was increased, but the circulating half-life was decreased with age [19]. Although the production of monocytes is increased with age, which would seem to imply increased numbers of monocytes in the blood, a decrease in the half-life of monocytes results in the total percentage of monocytes in the blood not changing. In addition to the skewed output of monocytes in blood, differentiation of HSCs is altered in BM. With aging, HSCs exhibit an intrinsic myeloid bias, thought to result in part due to increased IL-6 production [29,30].

Historically, monocytes were described by Ehrlich and Metchnikoff [24,39,40] where they were originally considered to be two populations based on CD14 and CD16 expression [24]. Currently in humans and non-human primates, there are thought to exist three populations that include classical (CD14^+^CD16^−^), non-classical (CD14^dim/lo^CD16^+^), and intermediate (CD14^+^CD16^+^) monocytes [41,42,43]. In general, it is considered that classical monocytes in humans—and their equivalents in mice—differentiate and may give rise to some tissue macrophages and dendritic cells (DC) where they are involved in inflammation and repair [40,42,44,45]. Early studies using ^3^H-Thymidine demonstrated that classical monocytes gave rise primarily to intermediate and non-classical monocytes [46]. We performed similar studies in normal and SIV-infected rhesus macaques using bromo-deoxyuridine (BrdU), a thymidine analogue taken up by myeloid precursor cells that can be detected in classical monocytes within 24 h, followed by intermediate monocytes (24 h later), and then non-classical monocytes (found 24 h later) [13,26]. These observations are similar to ^3^H-Thymidine studies in humans, which also suggest that intermediate and non-classical cells are a continuum of cells originating from classical monocytes that have undergone maturation and/or activation in blood [13,26,47]. Using BrdU uptake by monocytes, it was found that the magnitude of monocyte expansion in blood and output from BM are good indicators (better than plasma virus and CD4+ T cell nadir) of the rate of AIDS development and the severity of tissue pathogenesis in SIV-infected monkeys [13,19,26]. Moreover, Kuroda et al. found an age-dependent increase in the turnover of monocytes (discussed below). While the continuum of monocytes from classical to non-classical cells in blood is an attractive model, it likely is not absolute, where subsets may also arise from other non-BM compartments including the spleen [48]. Overall, the intermediate monocytes are thought to be an activated and more mature phenotype with potential antigen-presentation (APC) functions that are susceptible to HIV—and more so SIV—infection [42,45,49,50,51,52]. The percentage of intermediate monocytes increases with HIV and SIV infection and in fact correlates with the incidence of AIDS dementia complex (ADC)(pre ART) [53] and CVD with HIV and SIV infection [5,14,54]. Non-classical monocytes are thought to function as patrolling cells that interact with parenchymal tissue endothelium, including those involved with viral responses [40]. Changes in the proportion and absolute numbers of monocyte subsets are seen in HIV and SIV infection, HIV and SIV infection with aging (discussed below). Differences are also observed in the proportion of monocyte subsets between sexes with aging and HIV infection [40,55,56,57]. Overall, this general model helps when considering the immune phenotype of these cells in normal conditions, and with HIV and SIV infection, with HIV and SIV infection, and aging; it is clear there is a skew towards intermediate monocytes with both, as well as altered monocyte turnover in the blood. Lastly, while the exact signal(s) for monocyte production in BM are not defined, they are likely in part driven by monocyte/macrophage turnover and death in tissues, their response to monokines and trafficking chemokines, HIV and SIV viral antigens, and immune-activating factors in plasma, many of which are increased with aging.

It has been shown that the magnitude of BrdU+ monocytes in blood 24 h after BrdU pulse correlates with the rate of development of AIDS in SIV-infected monkeys where a greater magnitude of BrdU+-labeled monocytes correlates with increased histopathogenesis [13]. Interestingly, animals with 10% or more BrdU+ monocytes, detected as early as 21 days pi, are predicted to develop rapid AIDS [13,54]. Moreover, the higher the percentage of BrdU+ monocytes correlates with how rapidly animals succumb to AIDS and the severity of brain and cardiac pathology [13]. Additionally, sCD163—a biomarker for myeloid activation—in these monkeys positively correlated with the number of BrdU+ monocytes in a near-linear fashion [13]. Soluble CD163 in HIV-infected individuals correlates with the degree of neurologic dysfunction and non-calcified vulnerable plaque [17,58,59,60] suggesting that the degree of monocyte activation and expansion from bone marrow are good biomarkers of HIV and SIV comorbidities. It has been subsequently determined that sCD163 and another marker of activated monocytes, sCD14, both associated with HIV, all cause morbidity and mortality [61,62]. While the exact signals for this expansion of monocytes and increased sCD163 are not defined, Kuroda et al. showed that macrophage death and turnover in tissues are highly correlated [26]. It is interesting to note these markers also increase with aging, and aging with HIV. We recently found studying ART interruption, the percentage of BrdU+ monocytes also increases and decreases with plasma virus in SIV-infected macaques, suggesting that plasma virus also plays a role (Williams et al., unpublished data). Additionally, it is reasonable to postulate that monocyte activation and turnover occurs as these cells compensate for loss of the acquired immune response caused by CD4 T-memory destruction in early HIV and SIV infection, and perhaps aging [5]. Using math-modeling algorithms, Kuroda et al. showed that the expansion of monocyte production with SIV infection (based on BrdU) likely occurs with aging [63]. Again, while the signals for such production, in addition to replacing injured and dying macrophages in tissues, are not well-defined, they likely also include dying signals in tissues and tissue microenvironments, both of which are likely increased with aging.

## 3. Chronic Monocyte/Macrophage Activation, Inflammation, and “Inflamm-Aging”

Numerous groups have found that monocytes isolated from aged individuals have reduced activation, proliferation, or altered signaling, and delayed responses that result in increased cytokine production compared to young controls, which is further discussed below [64,65]. Additionally, at the transcriptomic level, there is an impact of aging on interferon signaling from all monocyte subsets following TLR stimulation [66,67]. Alterations in monocyte activation and cytokine production with aging and HIV infection result from dysregulation of HSC production and differentiation leading to chronic inflammation and older immunophenotypes, respectively [31,55]. Similar changes were found when comparing ART women with HIV versus viremic individuals where the latter were similar to controls who were 12 years older [55]. While overall it is tempting to think that ART may reverse immune activation and innate immune responses by decreasing the plasma and tissue virus levels, the length of time for which individuals have been on ART and when ART was initiated post-infection are important considerations. We studied the effect of durable ART on plasma sCD163 of individuals with acute infection (ART treatment after less than one year of HIV infection) and chronic infection (ART treatment after more than one year of HIV infection) and found that plasma sCD163 levels decreased after 3 months to levels found in uninfected controls [58]. This was in contrast to individuals who received ART after more than a year of HIV infection, where after 3 months of ART, sCD163 decreased, but not to levels found in acute infection and uninfected controls [58]. It is tempting to speculate that these differential responses are informative with regard to the preprogrammed genetics of the innate immune response to HIV, but they are also likely linked to the acquired immune response that include antibodies, CD4^+^ T-cell depletion by HIV, as well as CD8^+^ T-cell responses and HIV-repertoire breadth, all of which importantly are affected by HIV infection and aging [11]. Lastly, it is also important to consider the effects of ART alone, aside from the effects of decreasing plasma and tissue virus, that might also result in premature or accelerated aging, which further complicates the picture [32,68,69,70].

Signaling molecules, including TLRs, mannose receptors, and scavenging receptors, are altered with aging and may contribute to changes in immune populations. Changes in monocyte/macrophage immune phenotype with HIV and SIV infection and accelerated aging are likely also due to increased soluble factors in plasma, as well as the effects of translocation of bacteria across the gut, the metabolic effects of this, and coinfections including CMV with aging [71,72,73]. Additionally, the role of monocyte responses to surface-receptor ligation, including TLRs to LPS, endocab, and viral antigens should be considered. Recent studies showed changes in the cytokine production of monocytes isolated from aged individuals where following stimulation, there was an increase in TNF, IL-1, and IL-6 that is associated with non-AIDS-defined comorbidities with PLWHIV [74]. Monocytes isolated from aged individuals have increased cytokine production including IL-6, TNF-alpha, and IL-1 alpha and beta that drive alterations in circulating monocyte populations [33,75,76,77]. Hearps et al. found that plasma cytokines specific to monocyte activation—sCD163 and sCD14—increase with age [56]. Additionally, widespread alterations in expression and function of toll-like receptors are impaired with age and contribute to the dysregulation of innate immunity, specifically the production of myeloid cells [78]. It is unknown whether aging is the cause of alterations in signaling molecules, thus resulting in downstream effects on myeloid populations; or if inherent skewing of HSCs towards a myeloid shift results in changes of myeloid-signaling molecules and alterations to myeloid phenotype; or both; more research is necessary to determine this.

Changes in the proportion of monocyte subsets, their response to immune stimulation by cell-surface receptor ligation, and their function are reported in PLWHIV- and SIV-infected monkeys, but overall, extensive studies have not been carried out. Hearps et al. in a study of HIV infection versus healthy aging focused on immune phenotype and function in a cross-sectional study, and showed that young viremic HIV+ males had monocyte phenotypes similar to aged controls that included increased CD11b on CD14+CD16+ monocytes and downregulation of CD62L and CD115 [56]. Additionally, they found that innate immune markers sCD163 and CXCL10 increased in both young viremic and virologically suppressed individuals to levels similar to that seen in elderly controls. They also found decreased phagocytic function and telomere shortening compared to young controls [56]. Overall, there are similarities in men versus women in age-associated changes with increased proportions of intermediate and classical monocytes, but found women were phenotypically different than men [57].

## 4. Monocyte and Macrophage Traffic to Tissues with HIV, SIV, and Aging

Monocytes are produced in the bone marrow in response to colony-stimulating factors (CSF) and cytokines, and migrate towards specific sites of infection or injury. Extravasation of monocytes and subsequent differentiation into macrophages is beneficial in normal physiology to assist in clearing infections and promoting tissue repair. However, chronic accumulation of macrophages leads to inflammation and subsequent tissue damage. “Age-associated inflammation” is thought to be induced by increased inflammatory-activated monocytes, IL-6, and higher CCR2 production, which could lead to excess monocyte production and macrophage accumulation [76]. With age, there are increased plasma concentrations of inflammatory mediators (IL-6, IL-10, sICAM-1, sVCAM-1, and MCP-1) that promote monocyte extravasation and subsequent tissue inflammation [79]. Additionally, studies have focused on monocyte and macrophage cytokine dysregulation and NF-kB pathway activation (discussed below) as drivers of inflammation [34,80,81] in the context of HIV and SIV; and HIV, SIV, and aging.

The NF-kB pathway is a key mediator of cellular damage and stress response and is impaired with age [81]. A murine-bone-marrow study identified the largest fraction of cells with increased NF-kB expression as myeloid cells [82]. Activation of the NF-kB pathway in macrophages leads to the production of proinflammatory cytokines IL-6 and TNF-alpha. An analysis of monocytes from hospitalized individuals (elderly vs. young) showed higher NF-kB activation but reduced responses to TLR stimulation in elderly populations versus younger controls [81]. Importantly, these studies found that with age, continued stimulation of the NK-kB pathway may lead to immune tolerance and reduced response to subsequent stimuli. There are multiple stimulatory ligands and factors that signal through the NF-kB pathway, including TLRs via viral ligands, ROS stimulation, and—more recently identified—miRNA. Grants et al. attributes one driver of age-related inflammation and myeloid skewing as the loss of miRNA-146a, resulting in upregulation of the NF-kB pathway followed by upregulation of IL-6; however, more research is necessary to determine other contributors to age-related inflammation with chronic HIV infection [80].

## 5. Immunosenescence and Aging

Immunosenescence is the gradual decline of the functionality of the immune system commonly seen in conditions of chronic immune stimulation including HIV-1 infection, HIV-1 infection and aging, and aging alone [74,83]. Aging is thought to impair the ability of monocytes and macrophages to clear senescence cells due to reduced phagocytosis, chemotaxis, and accumulation of age-related inflammation. With aging, there is an increase in the expression of markers of senescence including P16^INK4a^, IL-1β, and NF-kB [35,84]. Additionally, adults with HIV+ had increased biomarkers of immunosenescence including TNF-alpha, IL-6, sCD163, and sCD14. The proinflammatory nature of nonclassical monocytes alone—including higher P16^INK4a^, NK-kB activity, and IL-1 alpha production—are considered markers of cellular senescence [36,84]. P16^INK4a^ expression is a marker of cellular senescence and is expressed by macrophages in response to external stimuli [36]. Myeloid-derived suppressor cells (MDSCs) are thought to be immunosuppressors that act primarily on T and B cells to drive a senescence phenotype. MDSCs are significantly increased with aging and may be potent influencers of immunosenescence; however, they are not well-understood and their role in aging and disease is unclear [83]. Studies have found that the elimination of senescent cells can extend one’s lifespan by inhibiting pathways of inflammation and senescence [37]; however, the exact role of myeloid cells in this or the effects of aging and immunosenescence in people living with HIV is not well-understood. 

## 6. Myeloid Mitochondria Dysfunction and Contribution to Immunosenescence

Dysfunction of macrophage mitochondria with aging can increase oxidative stress, susceptibility to DNA damage, and viral infections. This in turn promotes a senescent, proinflammatory microenvironment. Bauer and Fuente describe how the aging of macrophage mitochondria leads to dysfunction and expression of proinflammatory cytokines (IL-6, TNF-alpha, and IL-1), resulting in increased inflammation and an overall decline in physiological functions [38]. Specifically with aging, there is higher production of reactive oxygen species (ROS) from macrophages, mitochondrial dysfunction, increased tissue inflammation, and mutagen susceptibility [85]. Causes of mitochondrial dysfunction with aging are unknown, but believed to be associated with reduced mitochondrial respiratory capacity of myeloid cells [86]. Dysfunction of mitochondrial respiratory chain complexes in macrophages can result in mitochondrial membrane potential changes, which in turn can be used as a determinant of the contribution of a myeloid bias with aging [87]. In addition, there is increasing evidence of mitochondrial dysfunction in PLWHIV [88,89,90]. This includes adverse effects of nucleoside reverse-transcriptase inhibitors (NRTIs) that induce mutations in mitochondrial DNA (mtDNA) [90] and alterations to mtDNA that can affect mitochondrial morphology, energy, and ROS production [89]. Thus, age-related associations in reduced monocyte/macrophage mitochondrial function, the effect of ART reagents on mitochondria, and increased oxidative stress with aging and aging with HIV, may be significant factors leading to increased inflammation [91].

Myeloid cells can release ROS in circulation which might be amplified by the inherent HSCs’ myeloid shift with aging; however, their exact contribution with HIV and HIV and aging is not fully understood [92]. Transmission electron microscopy revealed age-associated changes in the overall number and density of mitochondria in macrophages of aged mice compared to young controls [93]. Additionally, macrophages use tryptophan to generate NAD+ de novo through the Kynurenine Pathway (KP). The loss of KP metabolites results in reduced production of NAD+ and disrupts macrophage mitochondrial respiration, leading to alterations in morphology and phagocytosis [94]. Overall, age-related and HIV-and-age-related changes in HSCs could affect the number of mitochondria in monocytes/macrophages, mitochondrial function, and regulation of ROS production [29,95]. Multiple mechanisms link oxidative stress and aging, including mitochondrial dysfunction, microRNA dysregulation, and cellular senescence.

## 7. Accelerated Aging with HIV Infection

Chronic immune stimulation caused by HIV-1 infection results in heightened production of pro-inflammatory mediators and likely contributes to accelerated aging [55,74]. Hearps et al. and others have found that individuals with HIV-1 have similar myeloid immune profiles to elderly individuals with increased CD16+ monocyte, and activation markers comparable to controls that are 10–14 years older [57]. Specifically, they found elevated CD16+ monocytes, sCD163, sCD14, and CXCL10 [55,57]. Analyzing CD14+ monocytes from young HIV+ individuals, Hearps et al. found impaired phagocytic function consistent with aged CD14+ monocytes [57]. In all, HIV infection may contribute to premature and accelerated aging of the immune system, likely due to chronic stimulation.

More recently, researchers investigating epigenetic patterns in DNA found increased age-associated methylation in PLWH that is consistent with premature aging [11,96,97]. DNA methylation in PLWHIV revealed an accelerated methylation pattern by approximately 14 years [96]. Individuals living with HIV-1 who had over four years of ART treatment did not have significant epigenetic age-related alterations; however, there were still patterns of increased age-associated methylation [98,99]. HIV-1 infection may accelerate aging, whereas ART treatment may slow age acceleration but does not stop it. Thus, while ART treatment extends the life expectancy of PLWH, there remains an increase in age-associated comorbidities such as HAND and cardiovascular disease due to overall dysregulation of systemic inflammation.

In a study comparing the mean telomere length of HSCs and monocytes from healthy controls (26 years old) and the elderly (65 years old), the telomeres in elderly HSCs and monocytes were significantly shorter than the younger controls [100]. Likewise, individuals with HIV-1 have significantly shorter telomeres in HSCs, monocytes (CD14+), and leukocytes than in uninfected aged-matched controls [57,101,102]. Additionally, researchers found an inverse relationship between myeloid activation (sCD163) and telomere length, indicating that immune activation may be the cause of telomere shortening [102]. Premature aging is present with apparent telomere shortening, even in young individuals with HIV and a study investigating children with HIV-1 (and treated with ART) revealed multiple age-associated alterations including higher percentages of activated cells (CD16+ monocytes), accelerated telomere shortening in HSCs, and higher percentages of senescent cells [101,103]. Although still not well-understood, studies on telomere length show that constant immune stimulation caused by HIV-1 infection may lead to accelerated aging. It is also possible that a lack or dysfunction of normal DNA repair within the BM in HSC may also contribute to telomere shortening.

## 8. Conclusions

Changes in the activation, proportion, migration, and phenotype of monocyte subsets are apparent with aging, and result in alterations of systemic immune physiology. Similarly, HIV and SIV viral RNA, even if not infectious, contribute to myeloid stimulation and activation. Due to immunosenescence and loss of the acquired immune responses to HIV and SIV, as well as other co-infections including CMV, it likely requires an expanded role of myeloid cells in blood and tissues to aid in pathogen patrol and controlling tissue injury and repair. It is known with aging that augmented hematopoietic production of BM HSC occurs that skews toward increased monocytes and likely preferential phenotypes seen with HIV and SIV and aging, as well as altered functional effects of stimulation of monocytes following the ligation of immune molecules, including TLRs and phagocytosis. This includes increased monocyte activation, increased inflammatory cytokines and markers of senescence, reduced monocyte/macrophage mitochondrial function, and increased oxidative stress, all of which are contributors to chronic inflammation.
